# Draft Genome Sequence of *Sporidiobolus salmonicolor* CBS 6832, a Red-Pigmented Basidiomycetous Yeast

**DOI:** 10.1128/genomeA.00444-15

**Published:** 2015-05-21

**Authors:** Marco A. Coelho, João M. G. C. F. Almeida, Chris Todd Hittinger, Paula Gonçalves

**Affiliations:** a UCIBIO, REQUIMTE, Departamento de Ciências da Vida, Faculdade de Ciências e Tecnologia, Universidade Nova de Lisboa, Caparica, Portugal; b Genome Center of Wisconsin, Laboratory of Genetics, DOE Great Lakes Bioenergy Research Center, Wisconsin Energy Institute, J. F. Crow Institute for the Study of Evolution, University of Wisconsin–Madison, Madison, Wisconsin, USA

## Abstract

We report the genome sequencing and annotation of the basidiomycetous red-pigmented yeast *Sporidiobolus salmonicolor* strain CBS 6832. The current assembly contains 395 scaffolds, for a total size of about 20.5 Mb and a G+C content of ~61.3%. The genome annotation predicts 5,147 putative protein-coding genes.

## GENOME ANNOUNCEMENT

The carotenoid-producing yeast *Sporidiobolus salmonicolor* belongs to the order *Sporidiobolales* (1), which is classified in the subphylum *Pucciniomycotina*, the earliest branching lineage of *Basidiomycota* (2). This species is recognized mainly as a phyllosphere yeast and is free-living and distributed worldwide. It has been recovered from a broad spectrum of substrates, including fresh and marine water, soil, and even clinical samples (3). This species has a research interest from the perspective of the evolution of sexual reproduction in basidiomycetes (4–6) and has the potential to serve as a natural source of carotenoids (7) for pharmaceutical, cosmetics, and food industries (8, 9). Here we report the genome sequencing of *S. salmonicolor* strain CBS 6832, isolated as a contaminant of a clinical sample (10), using a combination of Illumina and Pacific Biosciences (PacBio) single-molecule real-time (SMRT) sequencing technologies. For Illumina sequencing, 0.4-kb paired-end and 2- to 5-kb mate-pair libraries were generated and sequenced using the GAIIx and HiSeq 2000 platforms, respectively. For PacBio sequencing, a 10-kb SMRTbell library was generated and sequenced on a PacBio RS II platform. Illumina sequencing data were pre-processed by Trimmomatic version 0.32 (11). In short, adapter sequences were removed and low-quality bases were trimmed at the end of the reads and when the average quality was below a defined quality threshold (Phred score <20, using a sliding window approach). The *de novo* hybrid assembly of Illumina and PacBio data was performed using SPAdes version 3.1 assembler (12) with parameters “careful” and “K 35,55,65,77.” PacBio circular consensus sequences (CCS) were used as unpaired single reads, and contiguous long reads (CLR) were used for gap closure and repeat resolution. The accuracy of the resulting assembly was evaluated with REAPR (13), which uses read pairs mapped to the initial assembly to pinpoint misassemblies, such as scaffolding inaccuracies. The final draft assembly consists of 395 scaffolds (165 of which are above 500 bp), for a total size of 20,549,402 bp (*N*_50_, 538 kb) and a G+C content of about 61.3% as assessed by QUAST (14). Genes were predicted using Maker version 2.10 (15) with RepBase version 19.5 (16), SNAP, and Augustus trained on the *Rhodosporidium toruloides* NP11 model (PRJNA169538). Protein-coding sequences were annotated using the SIMAP database (17), as of late May 2014. Overall, we predicted 5,147 putative protein-coding genes. These encompass 798 superfamilies (18); 4,019 genes fall into 2,198 PANTHER families, of which 3,031 were annotated to the subfamily level (19). The most represented families are involved in transport, carbohydrate metabolism, regulation, and steroid metabolism. About 21% of the annotated proteins display a putative transmembrane region, 477 of which present four or more of these regions. A total of 1,031 genes exhibit coiled-coils signs in their products, suggesting involvement in protein–protein interactions (20). About 220 of the proteins present an identifiable signal peptide and are expected to be secreted (21, 22).

This genome will enable direct access to genes encoding enzymes with potential biotechnological applications and foster comparative genomics studies to elucidate fundamental biological processes, such as the evolution of sexual reproduction in fungi.

### Nucleotide sequence accession numbers.

The genome of *S. salmonicolor* strain CBS 6832 has been deposited in DDBJ/ENA/GenBank under the accession numbers CENE01000001 to CENE01000395. The version described in this paper is the first version.

## References

[1] Sampaio JP, Gadanho M, Bauer R, Weiss M 2003 Taxonomic studies in the Microbotryomycetidae: *Leucosporidium golubevii* sp. nov., *Leucosporidiella* gen. nov. and the new orders Leucosporidiales and Sporidiobolales. Mycol Prog 2:53–68. doi:10.1007/s11557-006-0044-5.

[2] Aime MC, Toome M, McLaughlin D 2014 The Pucciniomycotina, p 271–294. *In* McLaughlin DJ, Spatafora JW (ed), Systematics and evolution: the mycota, vol. VII, part A. Springer, Berlin.

[3] Sampaio JP 2011 *Sporidiobolus* Nyland, p 1549–1562. *In* Kurtzman CP, Fell JW, Boekhout T (ed), The yeasts: a taxonomic study, vol. 3, 5th ed., vol. 3, part Vb. Elsevier, Amsterdam.

[4] Coelho MA, Gonçalves P, Sampaio JP 2011 Evidence for maintenance of sex determinants but not of sexual stages in red yeasts, a group of early diverged basidiomycetes. BMC Evol Biol 11:249. doi:10.1186/1471-2148-11-249. 21880139 PMC3236058

[5] Coelho MA, Sampaio JP, Gonçalves P 2010 A deviation from the bipolar-tetrapolar mating paradigm in an early diverged basidiomycete. PLoS Genet 6:e1001052. doi:10.1371/journal.pgen.1001052. 20700437 PMC2916851

[6] Kües U, James TY, Heitman J 2011 Mating type in basidiomycetes: unipolar, bipolar, and tetrapolar patterns of sexuality, p 97–160. *In* Pöggele S, Wöstemeyer J (ed), Evolution of fungi and fungal-like organisms, vol. 6 Springer, Berlin.

[7] Colet R, Di Luccio M, Valduga E 2015 Fed-batch production of carotenoids by *Sporidiobolus salmonicolor* (CBS 2636): kinetic and stoichiometric parameters. Eur Food Res Technol 240:173–182. doi:10.1007/s00217-014-2318-5. 18841498

[8] Frengova GI, Beshkova DM 2009 Carotenoids from *Rhodotorula* and *Phaffia*: yeasts of biotechnological importance. J Ind Microbiol Biotechnol 36:163–180. doi:10.1007/s10295-008-0492-9. 18982370

[9] Schmidt-Dannert C, Umeno D, Arnold FH 2000 Molecular breeding of carotenoid biosynthetic pathways. Nat Biotechnol 18:750–753. doi:10.1038/77319. 10888843

[10] Misra VC, Randhawa HS 1976 *Sporobolomyces salmonicolor* var. *fischerii*, a new yeast. Arch Microbiol 108:141–143. doi:10.1007/BF00425104. 945046

[11] Bolger AM, Lohse M, Usadel B 2014 Trimmomatic: a flexible trimmer for Illumina sequence data. Bioinformatics 30:2114–2120 doi:10.1093/bioinformatics/btu170. 24695404 PMC4103590

[12] Bankevich A, Nurk S, Antipov D, Gurevich AA, Dvorkin M, Kulikov AS, Lesin VM, Nikolenko SI, Pham S, Prjibelski AD, Pyshkin AV, Sirotkin AV, Vyahhi N, Tesler G, Alekseyev MA, Pevzner PA 2012 SPAdes: a new genome assembly algorithm and its applications to single-cell sequencing. J Comput Biol 19:455–477. doi:10.1089/cmb.2012.0021. 22506599 PMC3342519

[13] Hunt M, Kikuchi T, Sanders M, Newbold C, Berriman M, Otto TD 2013 REAPR: a universal tool for genome assembly evaluation. Genome Biol 14:R47. doi:10.1186/gb-2013-14-5-r47. 23710727 PMC3798757

[14] Gurevich A, Saveliev V, Vyahhi N, Tesler G 2013 QUAST: quality assessment tool for genome assemblies. Bioinformatics 29:1072–1075. doi:10.1093/bioinformatics/btt086. 23422339 PMC3624806

[15] Holt C, Yandell M 2011 MAKER2: an annotation pipeline and genome-database management tool for second-generation genome projects. BMC Bioinformatics 12:491. doi:10.1186/1471-2105-12-491. 22192575 PMC3280279

[16] Jurka J, Kapitonov VV, Pavlicek A, Klonowski P, Kohany O, Walichiewicz J 2005 Repbase Update, a database of eukaryotic repetitive elements. Cytogenet Genome Res 110:462–467. doi:10.1159/000084979. 16093699

[17] Arnold R, Rattei T, Tischler P, Truong MD, Stümpflen V, Mewes W 2005 SIMAP—the similarity matrix of proteins. Bioinformatics 21(Suppl 2):ii42–ii46. doi:10.1093/bioinformatics/bti1107. 16204123

[18] Gough J, Karplus K, Hughey R, Chothia C 2001 Assignment of homology to genome sequences using a library of hidden Markov models that represent all proteins of known structure. J Mol Biol 313:903–919. doi:10.1006/jmbi.2001.5080. 11697912

[19] Thomas PD, Campbell MJ, Kejariwal A, Mi H, Karlak B, Daverman R, Diemer K, Muruganujan A, Narechania A 2003 PANTHER: a library of protein families and subfamilies indexed by function. Genome Res 13:2129–2141. doi:10.1101/gr.772403. 12952881 PMC403709

[20] Lupas A, Van Dyke M, Stock J 1991 Predicting coiled coils from protein sequences. Science 252:1162–1164. doi:10.1126/science.252.5009.1162. 2031185

[21] Käll L, Krogh A, Sonnhammer EL 2004 A combined transmembrane topology and signal peptide prediction method. J Mol Biol 338:1027–1036. doi:10.1016/j.jmb.2004.03.016. 15111065

[22] Petersen TN, Brunak S, von Heijne G, Nielsen H 2011 SignalP 4.0: discriminating signal peptides from transmembrane regions. Nat Methods 8:785–786. doi:10.1038/nmeth.1701. 21959131

